# Anti-Inflammatory Drugs Reduce the Risk of Hepatocellular Carcinoma Development

**DOI:** 10.5402/2011/390676

**Published:** 2011-07-07

**Authors:** Yasushi Rino, Kazuo Tarao

**Affiliations:** ^1^Department of Surgery, Yokohama City University, 3-9, Fukuura, Kanazawa-ku, Yokohama 236-0004, Japan; ^2^Tarao Clinic, 2-58-6 Futamatagawa, Asahi-ku, Yokohama 241-0821, Japan

## Abstract

Nowadays, patients with chronic hepatitis C in all countries are generally treated with interferon (IFN), and more than 50% of patients become HCV-RNA negative following PEG-IFN plus ribavirin therapy, but unfortunately, the IFN therapy is not effective in about 70% of patients with HCV-associated LC. In Japan, HCC actually develops in about 7% of those patients every year. A strategy for preventing HCC development other than IFN therapy is, therefore, urgently needed for those patients. We reported that the recurrence rate and the development of HCC was more rapid in the high serum ALT level (>80 IU) patients with HCV-associated LC. Sho-saiko-to, Juzen-taiho-to, and stronger-neo minophagen C are herbal medicines used in Japan to treat chronic viral liver diseases, and they work by reducing inflammatory processes and controlling ALT levels. Aggressive reduction therapy for ALT levels in HCV-LC patients could significantly prevent HCC development.

## 1. Introduction

Repeated inflammation and the resulting increased proliferation (mitotic activity) of tissue cells are correlated with the development of carcinoma, presumably by chromosomal instability, an increased rate of random mutations [1, 2], and promotion of tumor growth [3, 4]. There are many reported clinical instances that demonstrate the relationship between continuous inflammation and carcinogenesis: Helicobacter pylori infection and gastric cancer [5], ulcerative colitis and colorectal cancer [6], Clonorchis sinensis infection and cholangiocelluar carcinoma [7, 8], hepatitis C virus-(HCV-) associated liver cirrhosis (LC) and hepatocellular carcinoma (HCC) [9], and so on.

Considering the above findings, it is possible that the same mechanism is involved in the development of human HCC and that the development of HCC is accelerated by continuous inflammation in the liver of patients with HCV-associated LC. However, it is widely accepted that fibrosis may accelerate the development of HCC in HCV-associated liver diseases [10, 11].

## 2. Review

### 2.1. A Cut-Off Levels of ALT

In 1997 [12], we reported the relationship between the recurrence of HCC and the serum ALT level in hepatectomized patients with HCV-associated cirrhosis and HCC. Patients in Group A had no recurrence 3 years after surgery, and patients in Group B recurred during 1–3 years after surgery. The patients' serum ALT levels during this period were examined. In Group A, serum ALT generally showed sustained low levels <80 IU in 80% patients. In contrast, ALT levels in Group B showed several peaks or plateaus >80 IU in 81.2% patients. Moreover, with regard to the ALT levels, the recurrence rate of HCC in the hepatectomized patients with sustained low levels of ALT was 14.3% at 3 years and was significantly lower (*P* < 0.01) than that was 75.0% in those patients whose ALT levels showed several peaks or plateaus >80 IU. The importance of hepatocytic necrosis in the recurrence of HCC in hepatectomized patients with cirrhosis and HCC of HCV origin was demonstrated and the significance of subsiding hepatic necroinflammatory process in the prevention of HCC recurrence suggested. Because of this results, serum ALT level 80 IU was adopted as a cut-off level.

### 2.2. ALT Levels and Development of HCC

In 1999 [13], we reported association between high serum ALT levels and more rapid development and high incidence of HCC in patients with HCV-associated LC. In the paper, the correlation between the persistent elevation of serum ALT levels and the development of HCC was studied in patients with early-stage HCV-associated LC. Patients were subdivided into 2 groups according to their serum ALT levels: annual average serum ALT levels of Group A were persistently high (≥80 IU) and that of Group B was persistently low (<80 IU). HCC developed in 71.4% of patients in Group A compared with 25.0% in Group B over the observation period (*P* < 0.005). The 5-year rate of incidence of HCC in Group A was as high as 53.6% compared with only 7.1% in Group B (*P* < 0.001). The expected interval between the diagnosis of cirrhosis and the development of HCC was 6.0 ± 0.7 years (mean ± standard error (SE)) in Group A and 12.7 ± 1.2 years in Group B (*P* < 0.001). The results demonstrated that the development of HCC was more rapid in the high serum ALT level patients with HCV-associated LC.

### 2.3. ALT Levels and Recurrence of HCC

In 2000 [14], we investigated whether or not a high serum ALT level is associated with a more rapid recurrence of HCC in hepatectomized patients with HCV-associated LC and HCC. The hepatectomized patients with a single-nodule HCC had no histologic evidence of portal or hepatic vein invasion. They were subdivided into 2 groups according to their serum ALT levels: serum ALT levels showed several peaks or plateaus above 80 IU were designated as Group A, and serum ALT levels showed a sustained low level below 80 IU until first recurrence were designated as Group B. In Group A, HCC recurred within 3 years in 70.6% of patients. In contrast, it recurred in only 18.8% in Group B in the same period (*P* < 0.05). There was a significant difference (*P* = 0.0201) between the two groups in the cumulative nonrecurrence rate. The mean interval in recurrent patients between hepatectomy and the first recurrence in Group A (23.6 ± 2.8 months) (mean ± SE) was significantly (*P* < 0.02) shorter than that in Group B (49.3 ± 9.7 months). The expected interval between hepatectomy and recurrence was as short as 2.8 ± 0.5 years (mean ± SE) in Group A, compared with 5.8 ± 0.7 years in Group B (*P* < 0.05). These results showed that the recurrence of HCC was accelerated in the high serum ALT level patients with HCV-associated LC.

### 2.4. Multicentric Hepatocarcinogenesis and Sustained High ALT Levels

In 2002 [15], we investigated whether the persistent elevation of the serum ALT level is correlated with the multicentric development of HCC in patients with early-stage HCV-associated LC. Ninety-three patients with biopsy-proven HCV-associated LC (Child Stage A) were studied. They were subdivided into three groups according to their serum ALT level: Group A included 33 patients with annual average serum ALT levels that were persistently high (≥80 IU), Group B included 41 patients with annual average serum ALT levels that were persistently low (<80 IU), and Group C included 19 unclassified patients. The patients had been studied prospectively with frequent ultrasonography (US) and magnetic resonance imaging (MRI) or computed tomography (CT) for recurrence for >5 years. When the development of HCC was suspected, angiography, infusion into the hepatic artery, and lipiodol-CT were performed in all patients to determine the number of HCC nodules. In Group A, 27 patients (81.8%) developed HCC. Seventeen of 27 patients (63.0%) had multiple nodules. In contrast, in Group B, only 12 patients (29.3%) developed HCC, and only 1 of 12 patients (8.3%) had multiple nodules. There were significant differences between Group A and B in the incidence of developing HCC (*P* < 0.001) and developing multi-nodules (*P* = 0.006). In addition, among the male patients, the incidence of developing multiple HCC nodules in Group A (12 of 19 patients; 63.2%) was significantly higher (*P* < 0.05) compared with the incidence in Group B (0 of 6 patients; 0%). The same tendency was observed among female patients. These results showed a close correlation between multicentric hepatocarcinogenesis and sustained necroinflammation of the liver in patients with early-stage HCV-associated LC.

### 2.5. Low-ALT Levels and a Survival Advantage

In 2003 [16], we reported whether sustained alleviation of inflammation as monitored by serum ALT levels was associated with longer survival in hepatectomized HCC patients with HCV-associated LC. Thirty-four hepatectomized patients with HCV-associated LC and HCC as a single nodule, for whom more than 5 years had elapsed after the hepatectomy, were studied. They had no histologic evidence of portal or hepatic vein invasion. They were subdivided into two groups according to their serum ALT levels in the 2 years after hepatectomy: the low-ALT group comprised 13 patients whose serum ALT levels showed a sustained low level below 80 IU, and the high-ALT group comprised 21 patients whose serum ALT levels showed several peaks or plateaus above 80 IU. The patients had been followed up prospectively with frequent US and MRI or CT for recurrence for >5 years. The survival period, nonrecurrence interval, and number of recurrences were observed. Recurrences were treated with transcatheter chemoembolization in all cases. The cumulative survival rate in the low-ALT group was significantly better than that in the high-ALT group (*P* < 0.05). The 5-year survival in the low-ALT group was as high as 92.3% (12 of 13) compared with 33.3% (7 of 21) in the high-ALT group (*P* < 0  .05). The cumulative nonrecurrence rate in the low-ALT group was also significantly better than that in the high-ALT group (*P* < 0  .01). The survival period correlated well with the interval until the first recurrence (*r* = 0.545, *P* = 0.006). There was a tendency for the number of recurrences in the low-ALT group (1.5 ± 0.4, mean ± SE) to be fewer than that in the high-ALT group (2.2 ± 0.4) although this was not significant. Sustained alleviation of inflammation, as indicated by low-ALT levels, provides a survival advantage mainly due to the longer nonrecurrence interval, and possibly because of fewer recurrences, in hepatectomized HCC patients with HCV-associated LC.

### 2.6. Anti-Inflammatory Drugs

Previous our study suggested that suppression of the rise in ALT level by treatment using anti-inflammatory drugs may prolong the recurrent free interval and decrease the rate of development of HCC in patients with HCV-associated LC.

In 2006 [17], we reported the reduction therapy of serum ALT levels using herbal medicine. Sho-saiko-to [18], Juzen-taiho-to, and stronger-neo minophagen C (SNMC; glycyrrhizin [19]) are herbal medicines used in Japan to treat chronic viral liver diseases, and they work by reducing inflammatory processes and controlling ALT levels. Ursodeoxycholic acid (UDCA) and protoporphyrin are also used to suppress elevated ALT levels in some cases. Suzuki et al. [19] demonstrated a significant decrease in serum ALT levels obtained by intravenous injection of SNMC in patients with chronic hepatitis, and Hirayama et al. [20] showed a significant decrease in serum ALT levels obtained by oral administration of Sho-saiko-to in patients with chronic active hepatitis. Bellentani et al. [21] demonstrated a significant decrease in serum ALT levels obtained by oral administration of UDCA in the long-term treatment of patients with chronic hepatitis. To find a way to prevent the development of hepatocellular carcinoma (HCC) from hepatitis-C-virus-associated liver cirrhosis (HCV-LC), an analysis of the HCV-LC patients who had reduction therapy of ALT levels was performed. Seventy-four consecutive HCV-LC patients of Child Stage A were followed for >10 years for the development of HCC. They were divided into two groups: in group A, the reduction therapy for ALT levels is aggressively performed, and in group B, the reduction therapy was not performed aggressively. The patients were subdivided into 3 subgroups according to their serum ALT levels. In groups A and B, the high-ALT group was comprised, respectively, of 9 and 5 patients whose annual average serum ALT level was persistently high (≥80 IU); the low-ALT group was comprised of 19 and 20 patients whose annual average serum ALT level was persistently low (<80 IU), and the remaining 11 and 10 patients had an annual average serum ALT level which fluctuated and was unclassified (unclassified group). In group B, 65.7% of patients had developed HCC in 13 years, in contrast to only 41.0% in group A (*P* = 0.039). In group A, median HCC developing time was 12.8 years, in contract to only 3.8 years in group B (*P* = 0.0013). Multivariate analysis demonstrated that the mode of reduction therapy and ALT levels were the significant factors affecting HCC development. The chances of surviving for more than 10 years without developing HCC in the HCV-LC patients of Child Stage A were far more favorable in group A than in group B. These results suggest that aggressive reduction therapy for ALT levels in HCV-LC patients could significantly prevent HCC development.

### 2.7. High-ALT Levels Derived Carcinogens?

In 2009 [22], to assess retrospectively whether continuously high serum ALT levels (≥80 IU) in the first three successive years after the diagnosis of LC are predictive of a subsequent high incidence of HCC in patients with Child Stage A HCV-associated LC. The study comprised 132 HCV-associated LC (Child Stage A) patients who had not received interferon therapy but had been treated with anti-inflammatory agents [17]. At the end of a 3-year followup after the diagnosis of LC, the patients were subdivided into three groups according to their serum ALT levels, and the subsequent incidence of HCC was assessed. The cumulative incidence of HCC starting from 3 years after the diagnosis of LC in the continuously high-ALT group (annual average over 3 years always ≥80 IU; *n* = 41; Group A) was markedly higher than that in the continuously-low ALT group (always <80 IU; *n* = 48; Group B) (*P* < 0.005) during an observation period of 7.9 ± 3.7 years. The incidence of HCC in Group A was 11.8%/year. The odds ratios of developing HCC in Group A and Group C (mixed high and low ALT levels; *n* = 43) were 5.1-fold and 1.5-fold that of Group B, respectively. A multivariate analysis revealed that the ALT group was independently associated with HCC development. Continuously high ALT levels for three successive years following the diagnosis of LC can be predictive of a very high incidence of HCC in Child A HCV-associated LC patients. 

The final issue is the discussion of why the risk of developing HCC was increased so markedly in the continuously high-ALT group, as demonstrated in our previous studies. It is likely that genetic alterations accumulate rapidly as inflammation persists and that the multistep process of carcinogenesis or promotion of tumor growth progresses more rapidly in patients with continuously high ALT levels. In this respect, Ferenc et al. [23] demonstrated significant differences in the P53 expression between mildly, moderately, and severely inflamed biopsy samples in ulcerative colitis. 8-hydroxy-2′-deoxyguanosine (8-OHdG) is a promutagenic DNA lesion produced by oxygen (hydroxy) radicals [24, 25], and is known to be a parameter of genetic risk for hepatocarcinogenesis [26]. Furthermore, 8-OHdG was demonstrated to be involved in the initiation of rat liver hepatocarcinogenesis by low doses of N-nitrosodiethylamine [27]. Shimoda et al. [28] examined the levels of 8-OHdG in patients with chronic hepatitis, LC, and HCC and found that the OHdG level in liver affected by chronic hepatitis was significantly higher than that in normal liver and that the OHdG level in liver affected by LC also tended to be higher than that in normal liver. They also found a significant correlation between the OHdG content in noncancerous liver tissue and individual serum ALT levels and concluded that chronic inflammation in the liver might produce oxidative DNA damage, which would increase the risk of genomic alterations causing hepatocarcinogenesis. If high-grade inflammation persists in the liver for many years, as in the continuously high-ALT group of patients in our reports, the level of 8-OHdG might be high throughout the cirrhotic liver, resulting in the development of HCC.

### 2.8. The Reason that Our Aggressive Reduction Therapy of ALT Reduced HCC Development

Shiota et al. reported that Sho-Saiko-to prevents the development of HCC, and suppression of 8-OHdG formation may be the leading cause for inhibition of hepatocarcinogenesis by Sho-Saiko-to [26]. And Tsuchiya et al. reported that Juzen-taiho-to inhibited the development of liver tumors reduced oxidative DNA damage, inflammatory cell infiltration, and cytokine expression in mice [29]. These studies presented new important information on the anticancer effect of Sho-saiko-to and Juzen-taiho-to in human and provided mechanistic evidence that the protective effects are, at least in part, due to reduction in oxidant and cytokine production by Kupffer cells. These studies explain well the reason that our aggressive reduction therapy of ALT reduced HCC development. 

Nowadays, patients with chronic hepatitis C in all countries are generally treated with interferon (IFN), and more than 50% of patients become HCV-RNA negative following PEG-IFN plus ribavirin therapy, but unfortunately, the IFN therapy is not effective in about 70% of patients with HCV-associated LC. Moreover, patients with HCV-associated LC carry a high risk of HCC, and in Japan, HCC actually develops in about 7% of those patients every year [4]. A strategy for preventing HCC development other than IFN therapy is, therefore, urgently needed for those patients.

## 3. Conclusion

We demonstrated that if the serum ALT level was high (≥80 IU), then the risk of recurrence of HCC in hepatectomized patients and more rapid development of HCC increased markedly as compared with the continuously low-ALT group in Child A HCV-associated LC patients. However, prospective trials using therapeutic approaches to decrease ALT levels are necessary to confirm a positive impact of ALT reduction on the incidence of HCC in patients with HCV-associated LC. Our studies suggest that serum ALT levels in HCV-LC patients must be lowered to below 80 IU by anti-inflammatory drugs as soon as a diagnosis of LC is confirmed.
